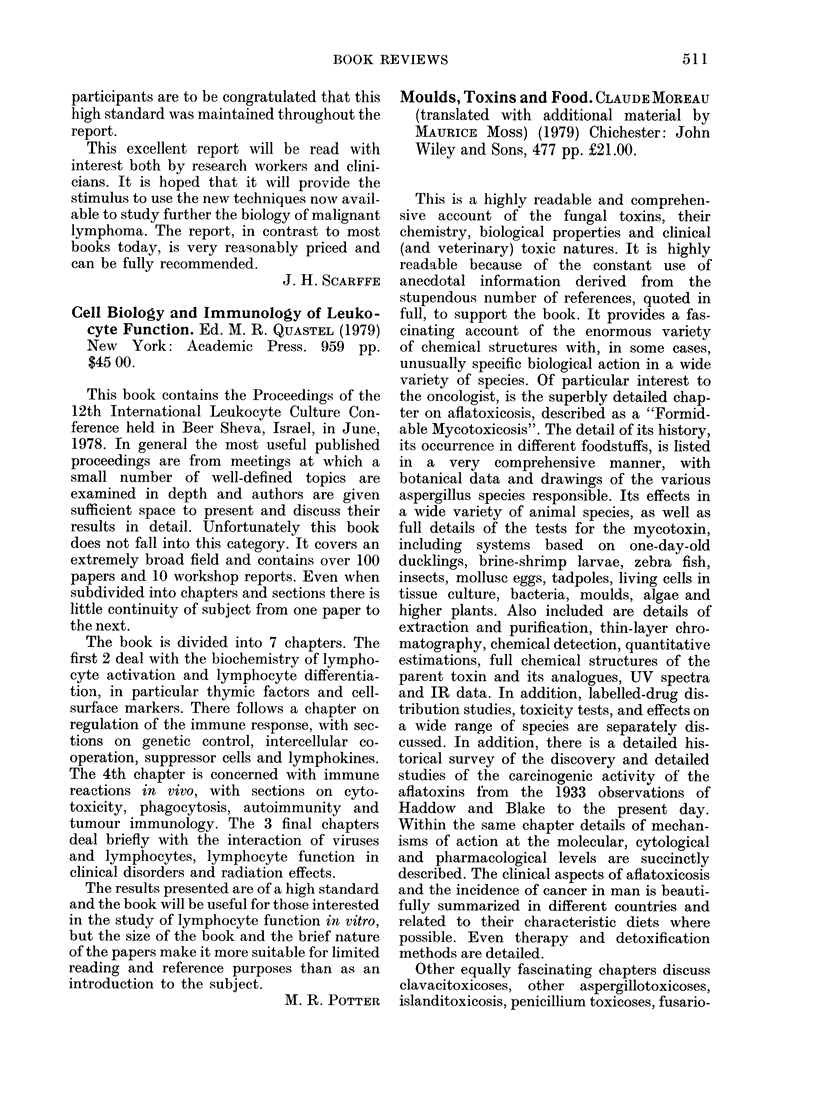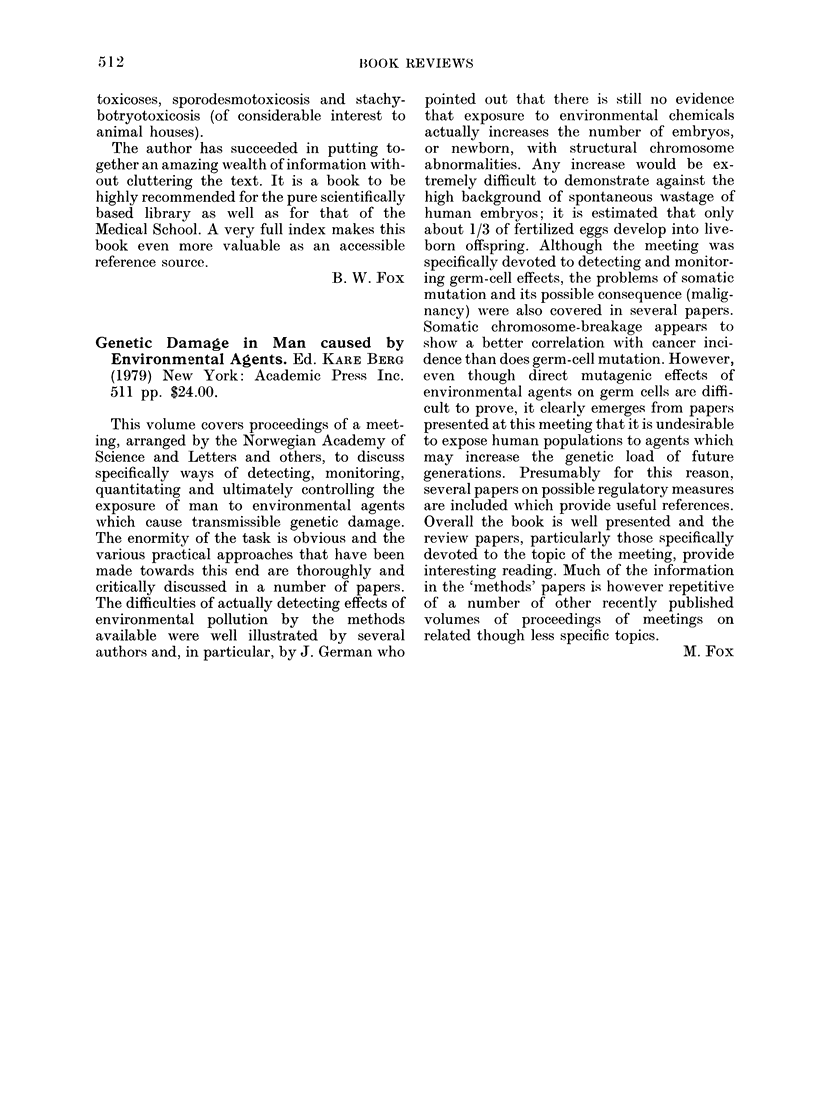# Moulds, Toxins and Food

**Published:** 1980-03

**Authors:** B. W. Fox


**Moulds, Toxins and Food.** CLAUDE MOREAU (translated with additional material by MAURICE MOSS) (1979) Chichester: John Wiley and Sons, 477 pp. £21.00.

This is a highly readable and comprehensive account of the fungal toxins, their chemistry, biological properties and clinical (and veterinary) toxic natures. It is highly readable because of the constant use of anecdotal information derived from the stupendous number of references, quoted in full, to support the book. It provides a fascinating account of the enormous variety of chemical structures with, in some cases, unusually specific biological action in a wide variety of species. Of particular interest to the oncologist, is the superbly detailed chapter on aflatoxicosis, described as a "Formidable Mycotoxicosis". The detail of its history, its occurrence in different foodstuffs, is listed in a very comprehensive manner, with botanical data and drawings of the various aspergillus species responsible. Its effects in a wide variety of animal species, as well as full details of the tests for the mycotoxin, including systems based on one-day-old ducklings, brine-shrimp larvae, zebra fish, insects, mollusc eggs, tadpoles, living cells in tissue culture, bacteria, moulds, algae and higher plants. Also included are details of extraction and purification, thin-layer chromatography, chemical detection, quantitative estimations, full chemical structures of the parent toxin and its analogues, UV spectra and IR data. In addition, labelled-drug distribution studies, toxicity tests, and effects on a wide range of species are separately discussed. In addition, there is a detailed historical survey of the discovery and detailed studies of the carcinogenic activity of the aflatoxins from the 1933 observations of Haddow and Blake to the present day. Within the same chapter details of mechanisms of action at the molecular, cytological and pharmacological levels are succinctly described. The clinical aspects of aflatoxicosis and the incidence of cancer in man is beautifully summarized in different countries and related to their characteristic diets where possible. Even therapy and detoxification methods are detailed.

Other equally fascinating chapters discuss clavacitoxicoses, other aspergillotoxicoses, islanditoxicosis, penicillium toxicoses, fusariotoxicoses, sporodesmotoxicosis and stachybotryotoxicosis (of considerable interest to animal houses).

The author has succeeded in putting together an amazing wealth of information without cluttering the text. It is a book to be highly recommended for the pure scientifically based library as well as for that of the Medical School. A very full index makes this book even more valuable as an accessible reference source.

B. W. Fox